# Flash Pulmonary Edema in a Young Adult With Unrecognized Critical Unicuspid Aortic Valve Stenosis

**DOI:** 10.7759/cureus.105122

**Published:** 2026-03-12

**Authors:** David J Connolly, Tanya Amal, Luke Man, Meghan Glaser, Richard Bloomingdale

**Affiliations:** 1 Internal Medicine, Beaumont Hospital, Royal Oak, USA; 2 Internal Medicine, Ascension St. Vincent Hospital, Indianapolis, USA; 3 Cardiology, Beaumont Hospital, Royal Oak, USA

**Keywords:** acute heart failure, aortic stenosis (as), bicuspid aortic valve, congenital heart defect, diastolic heart failure, echocardiography, flash pulmonary edema, pulmonary edema, unicuspid aortic valve

## Abstract

Unicuspid aortic valves (UAVs) are rare congenital anomalies associated with severe aortic stenosis (AS) in infancy or later adulthood. This is a case of a 22-year-old woman who presented with acute hypoxemic respiratory failure, which was initially attributed to multilobular pneumonia with acute respiratory distress syndrome. However, echocardiography revealed UAV with severe stenosis, suggesting cardiogenic pulmonary edema. The patient underwent successful surgical aortic valve replacement. This case highlights that the diagnosis and management of UAVs is tenuous: cases are prone to rapid decompensation, such as flash pulmonary edema, with acute hemodynamic changes, such as aggressive intravenous fluid administration. There currently exist no guidelines specific to UAVs, but diagnosis and management can be extrapolated from bicuspid aortic valves presenting with severe stenosis. Congenital valvulopathies should be considered in young adults with a heart murmur accompanied by exercise intolerance. Complex pre-existing cardiopulmonary hemodynamics necessitate a holistic approach to the management of acute conditions like pulmonary edema in this cohort.

## Introduction

Congenital malformations of the aortic valve (AV) occur as a result of failure of the aortic cusps to separate during embryogenesis, leading to a variable number of cusps, bicuspid (most common), unicuspid, or quadricuspid. Bicuspid aortic valve (BAV), the most common congenital cardiac malformation, typically presents as early-onset calcific aortic stenosis (AS) in the fifth to seventh decade. 

There are two unicuspid aortic valve (UAV) subtypes with differing presentations: acommissural and unicommissural. Acommissural UAV, which typically causes fatal or symptomatic AS in infancy, is characterized by three underdeveloped and fused commissures with a very narrow pinhole-like opening [1]. Unicommissural UAV is characterized by one normal and two underdeveloped and fused commissures with a slit-shaped opening [1]. Unicommissural valves have a larger valvular opening; thus, patients often present with symptomatic AS that mimics BAV, with or without concomitant aortic insufficiency (AI), in the third to fifth decade of life [2,3]. Symptoms typically include fatigue, dyspnea on exertion, and angina, often by age 30 [4]. In one cohort of 21 patients with UAVs, 91% had a known systolic murmur since childhood [4,5]. In addition, UAVs can be associated with aortic root dilatation, with an estimated prevalence of 20-60% [4,6]. UAVs are more prevalent in men (4:1 male-to-female ratio) [3] and occur with an estimated incidence of 0.02% in the general adult population. Incidence among those undergoing surgery for AS is approximately 5% (6% in men and 3% in women) [1,7]. 

Diagnosis of UAVs is difficult, as both transthoracic echocardiography (TTE) and transesophageal echocardiography (TEE) have poor sensitivity for UAVs (15% and 28%, respectively) [8]. However, specificity is more acceptable at 87% and 82% for TTE and TEE, respectively [8]. These patients often undergo surgical valve replacement due to severe AS regardless of AV morphology, and diagnosis is confirmed pathologically [8]. While no guidelines specific to the management of UAVs exist, including the most recent 2020 ACC/AHA valvular heart disease guidelines [9], practical management is very similar to that of the BAV. The difficulty in diagnosing UAVs is highlighted in the following case of a patient who did not quite fit the above cited typical demographics of UAVs and presented with hypoxic respiratory failure, initially misdiagnosed as multifocal pneumonia.

## Case presentation

History of present illness

A woman in her early 20s presented with chest pain, dyspnea, cough, and upper-respiratory tract infection-like symptoms. She was married one week prior in a large gathering. In the week following her wedding, she had been experiencing headache, cough, sore throat, emesis, and, eventually, exertional dyspnea. She was diagnosed with sepsis secondary to community-acquired pneumonia and was treated with ceftriaxone, azithromycin, and 2 L of intravenous normal saline. Her symptoms persisted, and she was transferred to a tertiary care center. She was initially vitally stable after transfer and was given 2.5 L of intravenous normal saline. On re-evaluation, the patient started to complain of worsened chest discomfort, diaphoresis, and shortness of breath. She had become tachycardic (HR 145 bpm) and hypoxemic (SpO2 70% on 4LNC). There were pink, frothy secretions from her mouth, and she was emergently intubated for airway protection. She required vasopressor support briefly for post-intubation hypotension (BP 70/40 mmHg). She was admitted to the medical intensive care unit for hemodynamic and ventilatory support. A grade III systolic murmur with radiation to the carotids was heard at the right upper sternal border. No peripheral edema was noted.

Past medical history

Past medical history was unremarkable; however, further questioning later revealed that the patient had poor exercise tolerance as a child compared to her peers.

Differential diagnosis

The initial presentation was most concerning for infectious etiology, given the acuity of the presentation and the recent large gathering. At the top of the initial differential, diagnosis was viral and/or bacterial pneumonia or infective endocarditis complicated by septic shock. Pulmonary embolism was also considered, given the acuity; however, this was quickly ruled out in the emergency department. Once it was known that the patient had pulmonary edema, acute respiratory distress syndrome (ARDS) was highly considered.

Investigations

**Figure 1:**
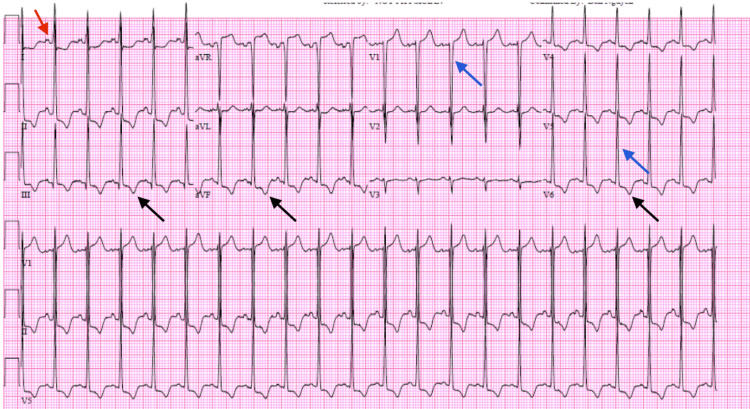
Initial ECG demonstrating signs of left ventricular hypertrophy (strain pattern T wave inversion in lateral precordial leads indicated by black arrows, deep S waves in lead V1 and tall R waves in V5-V6, indicated by blue arrows) and left atrial enlargement (biphasic P wave, indicated by the red arrow).

Initial electrocardiogram (ECG), shown in Figure 1, revealed sinus tachycardia with asymmetric T-wave inversions in the inferior and lateral precordial leads. There was evidence of left atrial enlargement and left ventricular hypertrophy by Sokolow-Lyon voltage criteria.

Initial labs (as shown in Table 1) were significant for neutrophilic leukocytosis (93% neutrophils), mildly elevated troponin I, elevated brain natriuretic peptide (BNP), mildly elevated creatinine kinase, and elevated inflammatory markers. Arterial blood gases showed hypoxemia and hypercarbia. Nine hours after initial presentation, troponin I had markedly increased from 300ng/L to 3,714ng/L with a two-hour repeat of 4,515ng/L.

**Table 1:** Initial laboratory values.

	Patient's	Reference Range
Leukocytes	24.1/mm^3^	3.3-10.7/mm^3^
Hemoglobin	11.6 g/dL	12.1-15.0 g/dL
Chloride	113 mmol/L	98-111 mmol/L
Bicarbonate	13 mmol/L	20-29 mmol/L
Brain natriuretic peptide	703 pg/mL	0-100 pg/mL
Creatinine kinase	495 U/L	30-150 U/L
C-reactive protein	236.1mg/L	< 8.0mg/L
Sedimentation rate	59mm/hr	0-20mm/hr
Arterial pH	7.21	7.35-7.45
PaCO2	51.5 mmHg	32-45 mmHg
PaO2	55.8 mmHg	80-100 mmHg
Troponin I @baseline	300 ng/L	<18n g/L
Troponin I @9hrs	3714 ng/L	<18 ng/L
Troponin I @11hr	4515 ng/L	<18 ng/L

Computed tomography pulmonary angiography was negative for pulmonary embolism but demonstrated bilateral consolidations and ground glass airspace opacities at the bilateral lung bases, left more severe than right.

Emergent bedside bronchoscopy revealed profoundly hyperemic, edematous trachea with a large amount of frothy pulmonary edema in the trachea and proximal main bronchi.

**Figure 2:**
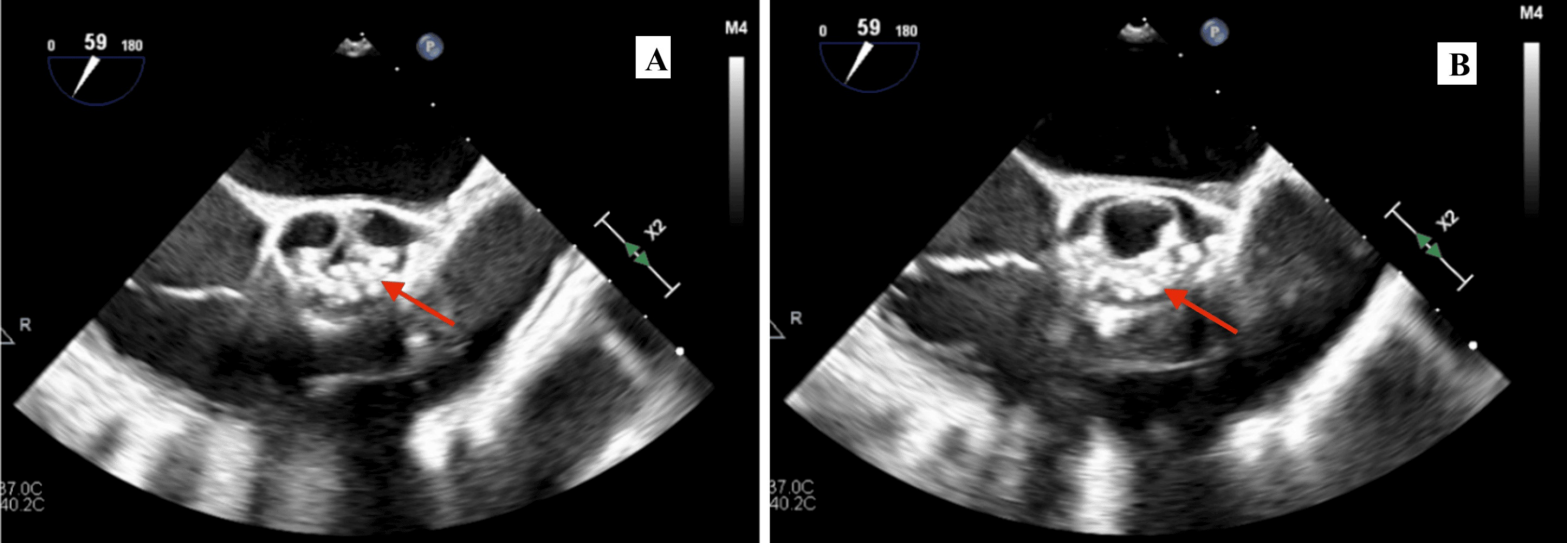
Transesophageal echocardiogram in the midesophageal short-axis view showing a severely calcified unicuspid aortic valve. The red arrows indicate the fused commissures.

TTE revealed an estimated left ventricular ejection fraction of 60%, moderately increased concentric LV wall thickness, grade I LV diastolic dysfunction, and mildly decreased RV systolic function by tricuspid annular plane systolic excursion. The AV was not well visualized but did appear to be significantly thickened with severe AS and mild insufficiency. The peak instantaneous gradient across the AV was 145 mmHg with a mean gradient of 82mmHg. The estimated aortic valve area (AVA) was 0.29 cm^2^ (by the continuity equation). TEE, shown in Figure 2, revealed an UAV with severe AS, mild AI, trace mitral and tricuspid regurgitation, and no pericardial effusion. The left atrium was mildly enlarged. There was no evidence of AV vegetation.

Infectious and rheumatologic workup remained unremarkable throughout the course of the admission.

Management

After being stabilized with intubation, mechanical ventilation, and vasopressor support, the patient was initially treated for septic shock from multilobar pneumonia complicated by ARDS with broad-spectrum antibiotics and corticosteroids. After the repeat high-sensitivity troponin I returned significantly elevated, she was treated for presumed cardiogenic pulmonary edema with intravenous diuretics.

After echocardiography showed a UAV, the patient was evaluated by cardiovascular surgery. By this time, she had been extubated and weaned off vasopressors. The patient opted for mechanical valve replacement. Intra-operatively, the UAV was visually confirmed. The aortic root and annulus were very small and were subsequently surgically enlarged and repaired with a Bovine pericardial patch. The UAV was removed and replaced with a 19mm On-X mechanical valve. Unfortunately, the native valve had become too fragmented during surgical extraction for UAV to be confirmed pathologically.

Outcome and follow-up

The patient tolerated the procedure well. Respiratory status initially remained tenuous; however, the patient was weaned to a nasal cannula by postoperative day 6. The patient was discharged in stable condition on postoperative day 9. The patient was seen in the outpatient follow-up six weeks after discharge, where she had been recovering well at home with improvement in functional status.

## Discussion

This case highlights the importance of recognizing the possibility of underlying congenital cardiac anomalies in young patients presenting with idiopathic respiratory and/or heart failure. This otherwise healthy young patient was presumed to have sepsis secondary to pneumonia. The standard treatment for this condition, aggressive intravenous fluids, turned out to be detrimental in this young patient with an unrecognized UAV with severe AS. As evidenced by this patient’s symptomatology, patients with UAV may decompensate rapidly. Flash pulmonary edema, particularly in the setting of severe AS, occurs as a result of acutely increased LVEDP, typically triggered by myocardial ischemia, increased preload, and/or increased afterload [10]. 

Increased afterload

With an AVA of 0.29 cm^2^, an aortic valve maximum velocity of 6.03m/s, a stroke volume index of 19.6mL/min^2^, and a mean aortic gradient of 82 mmHg, our patient’s AS can be classified as severe high gradient AS. With this chronically elevated afterload, further acute increases in afterload, such as a hypertensive emergency, could lead to flash pulmonary edema. However, in this case, systemic blood pressure was normal.

Myocardial ischemia/injury

In a young patient with low risk of coronary artery disease and presumably normal coronary arteries, ischemia causing LV dysfunction would be unlikely. However, this patient did develop myocardial injury as evidenced by elevated troponin. Coronary angiography was not performed, which limits the exclusion of myocardial injury with non-obstructive coronary arteries. However, assuming these limitations, the myocardial injury was most likely due to a supply-demand mismatch due to iatrogenically increased preload. 

Increased preload

Our patient received a total of 4.5L in IV fluids between two emergency departments. In the setting of chronically elevated afterload, this acute increase in preload was the most likely predisposing culprit of left ventricular dysfunction. No invasive hemodynamic values were available in this case; however, estimated values on transthoracic echocardiography can be used for extrapolation. Left-sided volume overload was evident with a left atrial volume index of 40 mL/m^2^ and bowing of the interatrial septum.

## Conclusions

This case provides a novel insight into a potential clinical manifestation of UAVs when unrecognized. In this case of severe UAV stenosis, overwhelming flash pulmonary edema resulted from an acute iatrogenic increase in preload (intravenous fluids), leading to LV dysfunction and supply-demand mismatch. Caution should be exerted with intravenous fluid management in such patients who may have congenital aortic valve malformations.

This case additionally demonstrates the importance of maintaining a wide differential diagnosis when clinical status unexpectedly deteriorates despite seemingly appropriate initial management. Typical demographic trends should be broadened with patients presenting with unexplained symptoms to prevent delayed or missed diagnoses. When considering causes of idiopathic heart failure in patients of all ages, valvular abnormalities should be included in the differential diagnosis.

## References

[1] von Stumm M, Sequeira-Gross T, Petersen J, Naito S, Müller L, Sinning C, Girdauskas E (2021). Narrative review of the contemporary surgical treatment of unicuspid aortic valve disease. Cardiovasc Diagn Ther.

[2] Lindman BR, Bonow RO, Otto CM Aortic valve stenosis. Braunwald's Heart Disease: A Textbook of Cardiovascular Medicine.

[3] Abdi SQ, Hassani K (2023). The study of the relationship between unicuspid aortic valve insufficiency and heart disease by fluid-structure interaction modeling. Biomedical Engineering Adv.

[4] Mookadam F, Thota VR, Garcia-Lopez AM, Emani UR, Alharthi MS, Zamorano J, Khandheria BK (2010). Unicuspid aortic valve in adults: a systematic review. J Heart Valve Dis.

[5] Novaro GM, Mishra M, Griffin BP (2003). Incidence and echocardiographic features of congenital unicuspid aortic valve in an adult population. J Heart Valve Dis.

[6] Zhu Y, Roselli EE, Idrees JJ (2016). Outcomes after operations for unicuspid aortic valve with or without ascending repair in adults. Ann Thorac Surg.

[7] Roberts WC, Ko JM (2005). Frequency by decades of unicuspid, bicuspid, and tricuspid aortic valves in adults having isolated aortic valve replacement for aortic stenosis, with or without associated aortic regurgitation. Circulation.

[8] Slostad BD, Witt CM, O'Leary PW, Maleszewski JJ, Scott CG, Dearani JA, Pellikka PA (2019). Diagnostic accuracy of echocardiography and intraoperative surgical inspection of the unicuspid aortic valve. Am J Cardiol.

[9] Otto CM, Nishimura RA, Bonow RO (2021). 2020 ACC/AHA Guideline for the management of patients with valvular heart disease: a report of the American College of Cardiology/American Heart Association Joint Committee on Clinical Practice Guidelines. J Am Coll Cardiol.

[10] Rimoldi SF, Yuzefpolskaya M, Allemann Y, Messerli F (2009). Flash pulmonary edema. Prog Cardiovasc Dis.

